# Nanoformulations as a modern form of biofungicide

**DOI:** 10.1007/s40201-020-00445-4

**Published:** 2020-01-31

**Authors:** Małgorzata Miastkowska, Alicja Michalczyk, Katarzyna Figacz, Elżbieta Sikora

**Affiliations:** 1 Faculty of Chemical Engineering and Technology, Institute of Organic Chemistry and Technology, Cracow University of Technology, Kraków, Poland; 2 Lukasiewicz - Research Network-Institute of Industrial Organic Chemistry, Warsaw, Poland

**Keywords:** Manuka oil, Cinnamon oil, Thyme oil, Tea tree oil, Nanoemulsions, Biofungicide

## Abstract

**Purpose:**

The aim of this study was to elaborate new forms of biofungicide formulations which could increase biological activity of essential oil against various strains of pathogenic fungi of plants, dermatophytes, and molds.

**Methods:**

The nanoemulsions containing four various essential oils (cinnamon, thyme, manuka, and tea tree oil) were obtained by using the low-energy (PIC) and the high-energy emulsification methods (ultrasonification). The physicochemical properties and activity of prepared systems against strains of pathogenic fungi of plants (*F. culmorum, Ph. cactorum*), dermatophytes (T*. mentagrophytes M. gypseum*) and molds (*S. brevicaulis, A. niger*) were examined. Fungicidal activity was tested by the method of linear growth of mycelium on an agar medium. Macroemulsions containing the oils and the pure essential oils were used as comparative samples.

**Results:**

It was found that nanoemulsions prepared by ultrasonification showed excellent fungicidal activity compared to pure oils and macroemulsions. Among others, the manuka oil nanoformulations showed the highest activity against the tested fungi.

**Conclusions:**

Nanoemulsions can be applied as the effective carriers of essential oils. They allow the reduction of the concentration of the bioactive oils while maintaining biological activity. The obtained nanosystems can be applied as safe, biodegradable, eco-friendly antifungal products in pharmaceutical, cosmetic, and agrochemical industries as they increase the biological activity of the tested oils against various type of fungi.

## Introduction

Pathogenic fungi cause infections in humans, plants, and animals. Among the species of fungi that can cause mycoses of plants, the most serious threat are the representatives of *Fusarium* and *Alternaria* [1–3]. However, the mycosis of the skin and its appendages in humans and animals is caused by dermatophytes (*Trichophyton, Microsporum, Epidermophyton*), yeast-like fungus (*Candida, Pitorosporum, Cryptococcus*) and mold fungus (*Aspergillus, Scopulariopsis*) [4].

When it comes to agrochemical industry, the synthetic fungicide application, although effective, results in several negative effects on the environment, such as: acute toxicity, long degradation periods, accumulation in food chain, and toxicity to non-target organisms, resulting in environmental, ecological, and health problems [5]. In the case of dermatophytoses, the increase of fungal resistance to traditional drugs is observed. Moreover, the majority of the available antifungal drugs show only fungistatic activity [6].

For this reason, pharmaceutical, cosmetic, and agrochemical nanoemulsions still looking for active ingredients of natural origin, which show antimicrobial activity.

The uses of plant-derived products as disease control agents have been studied since they tend to have low mammalian toxicity, low environmental effects, high degradability, multiple mechanisms of action, and fewer incidences of the numerous side effects often associated with synthetic chemicals [5, 7–10]. Plant essential oils may provide potential alternatives to currently used phytopathogenic fungi control agents, as a rich source of bioactive components. Many essential oils such as *Thymus vulgaris* oil [8, 11], *Melaleuca alternifolia* (tea tree) oil [12], clove oil [11, 13, 14], cinnamon oil [11, 15], manuka oil [11, 16], and citronella oils [17] have been reported to exhibit antifungal activities. Radwan et al. [14] studied the effect of thyme, clove, and cinnamon essential oils on *Candida albicans* and *Aspergillus* species. Their studies revealed that thyme oil inhibited the growth of different fungal isolates in 100% at concentrations of 0.25, 0.5 and 1%. Clove and cinnamon oils inhibited the growth of different fungal isolates at a concentration of 6% with the same inhibition rate (100%). Also, Elgayyar et al. [18] and Omidbeygi et al. [13] found that thyme essential oil was one of the most effective inhibitors of antifungal activity. On the other hand, Pires et al. [19] investigated the anticandidal activity of 16 essential oils and found that cinnamon was the most active essential oil showing anticandidal activity.

As reported in the relevant literature, the antimicrobial activity of essential oils is explained by their hydrophobicity. It decreases with the decrease of volatile constituents: phenols (highest activity) > alcohols > aldehydes >ketones > ethers > hydrocarbons [8, 20]. The main components of the essential oils are phenols (e.g. thymol, carvacrol). These substances interact strongly with the lipids of the fungi cell membrane and increase the membrane permeability, thus disturbing the original cell structure and, in consequence, leading to the cells’ deaths [14, 21–24].

It should be noted that apart from the active substances themselves, also the form of their carriers plays an important role in the efficiency of such products. Therefore, the development of bio-based nanomaterials, which are effective, safe, and environmentally friendly, is very important [17, 25]. Among other substances, nanoemulsions have many advantages. Due to small droplet size, large surface area, sustained release over time of active compounds, and stability against sedimentation, they can act as safe, target specific, biodegradable, and eco-friendly biopesticides [17, 26].

In recent years, there has been a growing interest in the nanoemulsions which are based on essential oils and which could serve as modern antimicrobial products. Larvicidal activity of *Rosmarinus officinalis* essential oil nanoemulsion was studied by Duarte et al. [27]. D-limonene based nanoemulsion was prepared by Li and Lu [28]. Wang et al. [26] prepared nanoemulsions containing water-insoluble pesticide, β-cypermethrin. Yildirim et al. [29] investigated the antimicrobial activity of Cinnamon oil based nanoemulsions against *E.coli* as the model microorganism. The antimicrobial activity (*Staphylococcus aureus, Escherichia coli, Pseudomonas Aaeruginosa, Enterococcus faecalis*) of the nanoemulsions with citral essential oil were analyzed by Lu et al. [30]. The effect of a *Salvia multicaulis* essential oil-containing nanoemulsion against food-borne pathogens was investigated by Gharenaghadeh et al. [31].

It is worth noting that antifungal nanoemulsions are much less reported in the literature. Abd-Elsalam and Khokhlov [25] studied antifungal activity of a eugenol oil nanoemulsion against *Fusarium oxysporum* f. sp. *vasinfectum* in cottonseeds. The antifungal activity of the nanoemulsions of neem and citronella oils against phytopathogenic fungi, *Rhizoctonia solani* and *Sclerotium rolfsii*, was investigated by Mohamed Ali et al. [17]. In most of the cases, the authors note that the antimicrobial activity of the essential oil-based nanoemulsions is much stronger than in free essential oils. The mechanism of the antimicrobial activity of the essential oil-based nanoemulsion was summarized by Donsi and Ferrari [24]. They suggested that antimicrobial efficacy of the nanoemulsions with an essential oil strongly depends on the kind of the essential oil components, tested microbial strains, the nanoemulsion composition, and internal phase droplet size. Due to small droplet size and increased surface area, the nanoemulsions influence the transport of the essential oils to the microbial cell membrane and their interaction with the multiple molecular sites at the cell membrane.

The aim of this study is to create new carrier forms of biofungicide formulations which could increase the biological activity of the essential oils such as cinnamon, thyme, manuka, and tea tree oil against strains of pathogenic fungi of plants (*F. culmorum, Ph. cactorum*), dermatophytes (T*. mentagrophytes M. gypseum*), and molds (*S. brevicaulis, A. niger*).

## Materials and methods

### Materials

The nanoemulsions containing four different essential oils (cinnamon oil, thyme oil, manuka oil, tea tree oil) stabilized by Polysorbate 80 were obtained. In the case of macroemulsions, the oil phase consists of the mixture of Crodamol GTCC and the essential oil. The cetyl alcohol and carbomer were added as stabilizers and also rheology modifiers to improve the stability of the conventional emulsions. Distilled water was used as the aqueous phase in all emulsions. The raw materials used in the study are shown in Table 1.

**Table 1:** Ingredients used in macro- and nanoemulsion formulation

Raw material	INCI name	Producer
Tween 80	Polysorbate 80	Sigma- Aldrich
Crodamol GTCC	Caprylic/Capric Triglyceride	Croda
Cetyl alcohol	Cetyl Alcohol	POCH
Carbopol Ultrez 30	Carbomer	Lubrizol
Cinnamon oil	*Cinnamomum zeylanicum* Oil	DrBeta
Tea tree oil	*Melaleuca alternifolia* Leaf Oil	DrBeta
Manuka oil	*Lystospermum scoparium* Oil	DrBeta
Thyme oil	*Thymus vulgaris* oil	ETJA

### Preparation of nanoemulsions

The nanoemulsions were obtained by using the low-energy phase inversion composition method (PIC) and high-energy emulsification methods (ultrasonification).

In the case of nanoemulsions obtained by PIC, the water phase was added drop by drop (at constant temperature T = 25 ± 1 °C) to the homogenous mixture of surfactant and oil, previously combined in various weight ratios (1:3; 1:4; 1:5, 1:7; 1:8, 1:9; 1.5:18.5; 2.5:17.5). The system was obtained under continuous stirring with Vortex Genius shaker (IKA).

In the ultrasonic emulsification method, the first step was the preparation of the pre-emulsion by combining the aqueous phase with the mixture of the essential oil: surfactant (1:1 weight ratio) under magnetic stirring at T = 25 °C. Then, the coarse emulsion was processed with a probe-type sonicator (UP200 Ht, Hielscher) for 1 min with 50% amplitude at 20 W.

### Preparation of macroemulsion

The aqueous-phase ingredients and the oil-phase ingredients (without the essential oil) were placed into separate beakers and heated up to T = 70 °C in a water bath. The emulsification process was carried out with a mechanical stirrer IKA EUROSTAR 20 (v = 500 rpm). The essential oil was added to the emulsion after cooling down to below 40 °C and then the system was being continuously stirred and cooled to the room temperature.

### Determination of the particle size of nanoemulsions

The mean droplet diameter and polydispersity index of the nanoemulsions were measured using a Dynamic Light Scattering (DLS) method (Zetasizer Nano ZS, Malvern Instruments, Malvern, UK) at 25 °C. The scattering angle was 173°. The analysis was performed three times (*n* = 3) to determine the mean values of the droplet size and standard deviation.

### Determination of the particle size of macroemulsions

The morphology of the macroemulsion was analyzed with an optical microscope (B1 Advanced Series, Motic). The mean value of the droplet diameter was an average of 200 measurements (*n* = 200).

### Antifungal activity evaluation

The activity of the essential oils and the obtained formulations (the nanoemulsions and macroemulsion) based on the oils was evaluated against the following fungal species: *Fusarium culmorum* (Smith) Saccardo, *Phytophthora cactorum* (Lebert et Cohn) Schroeter (plant pathogenic fungi), *Trichophyton mentagrophytes* ATCC 9533, *Microsporum gypseum* ATCC 6231 (dermatophytes), *Aspergillus niger* ATCC 16404, *Scopulariopsis brevicaulis* ATCC 36840 (filamentous fungi). The dermatophytes and filamentous fungi were supplied by the American Type Culture Collection (ATCC), USA. The plant pathogenic fungi were obtained from the Department of Phytopathology and Entomology, University of Warmia and Mazury in Olsztyn, Poland. The experiments were carried out according to a slightly modified standard method used in the laboratory investigations of fungicides [32, 33]. The test was performed on the Sabouraud Dextrose Agar (SDA) substrate in the concentration range from 2.5% to 0.5% (the essential oils) and 0.5% (macroemulsions). Acetone solutions of the test compounds were applied in an amount of 1 ml to the surface of agar-solidified medium on Petri dishes and then uniformly distributed and left to evaporate the solvent under aseptic conditions. Next, the plates were loaded with mycelial discs (5 mm diameter) cut from homogeneous 5–7-day-old cultures of the test fungi. As control acetone was applied to the agar medium followed by the mycelial discs after acetone had evaporated. The test was repeated three times. The dishes were incubated in the dark at 25 ± 1 °C (plant pathogenic fungi, dermatophytes) and 28 °C (filamentous fungi) for a period of 3–7 days. After the incubation, the diameters of the cultures were measured. The growth of the fungi on media containing the tested substance was compared to the control ones. The percentage of fungal growth inhibition (I%) was calculated according to the Abbott’s formula:

$$ \mathrm{I}\left(\%\right)=\frac{dc- dt}{dc}\ast 100 $$

dc: the diameter of the mycelium growth in the control plate (mm).

dt: the diameter of the mycelium growth in the experimental dish (mm).

### Statistical analysis

All data concerning the mean droplet size of nanoemulsions and biological activity of antifungal agents were presented as a mean of three different experiments ± SD. Differences between the calculated means of each individual group were determined by one-way ANOVA tests, using the statistical software Statistica Version 12 StatSoft Company, Cracow, Poland. A value of *p* < 0.05 was considered statistically significant.

## Results

### Composition of the obtained formulations

The macro- and nanoemulsion formulations, containing essential oils (cinnamon oil, thyme oil, manuka oil, tea tree oil) with the concentration range from 1.5 to 2.5% were obtained (Table 2). The nanoformulations were prepared by two methods: PIC method and ultrasonification (US).

**Table 2:** Composition of the formulations

Sample name	Ingredients [%]									
	OC	OT	OM	OH	T80	Aqua	GTCC	CA	Cb30	Method
NE-OC-2.5 -PIC	2.5	–	–	–	17.5	80	–	–	–	PIC
NE-OC-1.5 -PIC	1.5	–	–	–	18.5	80	–	–	–	PIC
NE-OT-1.5-PIC	–	1.5	–	–	18.5	80	–	–	–	PIC
NE-OM-1.5-PIC	–	–	1.5	–	18.5	80	–	–	–	PIC
NE-OH-2.0-PIC	–	–	–	2.0	18.0	80	–	–	–	PIC
NE-OH-1.5-PIC	–	–	–	1.5	18.5	80	–	–	–	PIC
NE-OC-2.5-US	2.5	–	–	–	2.5	95				US
NE-OC-1.5-US	1.5	–	–	–	1.5	97	–	–	–	US
NE-OT-1.5-US	–	1.5	–	–	1.5	97				US
NE-OM-1.5-US	–	–	1.5	–	1.5	97	–	–	–	US
NE-OH-2-US	–	–	–	2.0	2.0	96				US
NE-OH-1.5-US	–	–	–	1.5	1.5	97	–	–	–	US
ME-OC-2.5	2.5	–	–	–	6	80.5	5.5	6	0.5	–
ME-OT-1.5	–	1.5	–	–	6	80.5	5.5	6	0.5	–
ME-OM-1.5	–	–	1.5	–	6	80.5	5.5	6	0.5	–
ME-OH-2.0	–	–	–	2.0	6	80.5	5.5	6	0.5	–

*ME* macroemulsion; *NE* nanoemulsion; *OC* cinnamon oil; *OT* thyme oil; *OM* manuka oil; *OH* tea tree oil; *T80* Tween 80; *GTCC* Caprylic/capric triglyceride; *CA* cetyl alcohol; *Cb30* Carbopol 30; *PIC* Phase inversion method; *US* Ultrasound method

### Physicochemical properties of the obtained formulations

**Fig. 1:**
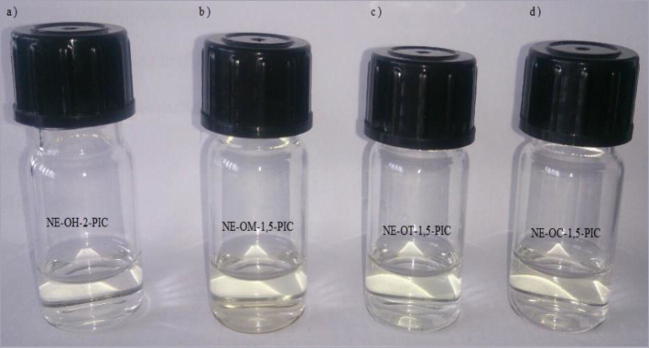
Macroscopic picture of the nanoemulsions obtained by the PIC method

**Fig. 2:**
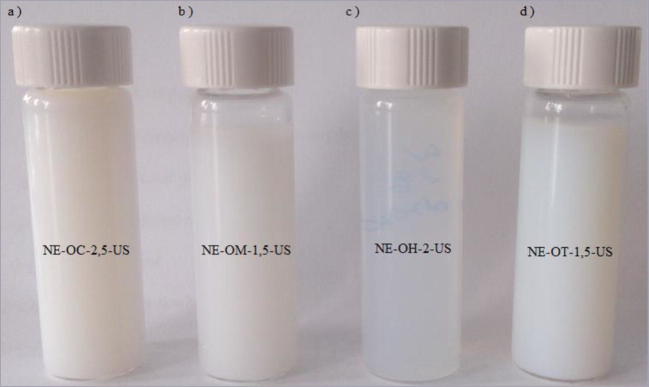
Macroscopic picture of the nanoemulsions obtained by the US method

The mean droplet diameter (Z-Ave) and polydispersity index (PDI) of the nanoemulsions, at the day of preparation and after 24 h of storage, are shown in Tables 3 and 4. In case of the formulations obtained by the PIC method all samples were transparent (Fig. 1). The formulations prepared by ultrasonification were milky/translucent (Fig. 2). The maximum concentration of the essential oil incorporated to the formulation obtained by low energy emulsification method was 2.5% (only in case of the cinnamon oil). For this reason, the concentration of the essential oils incorporated in the nanoemulsions obtained by the high energy emulsification method and macroemulsions was analogous to their concentration in the nanoformulations obtained by the PIC method.

**Table 3:** The mean droplet size of the nanoemulsions prepared by the PIC method, on the day of preparation and after 24 h of storage, *n* = 3 ± S.D.)

Sample name	t = 0		t = 24 h	
	Z-Ave [nm]	PDI	Z-Ave [nm]	PDI
NE-OC-2.5-PIC	14.3 ± 0.2	0.331 ± 0.012	14.7 ± 0.5	0.327 ± 0.017
NE-OC-1.5-PIC	14.5 ± 0.1	0.404 ± 0.061	68.53 ± 0.2	0.315 ± 0.090
NE-OH-2-PIC	16.2 ± 0.3	0.523 ± 0.060	15 ± 2	0.498 ± 0.055
NE-OH-1.5-PIC	16.4 ± 0.2	0.530 ± 0.033	40 ± 5	0.473 ± 0.040
NE-OM-1.5-PIC	28 ± 1.0	0.356 ± 0.016	12 ± 1	0.256 ± 0.012
NE-OT-1.5-PIC	15.2 ± 0.8	0.444 ± 0.009	14.8 ± 2.8	0.407 ± 0.015

**Table 4:** The mean droplet size of the nanoemulsions prepared by the ultrasound method, on the day of preparation and after 24 h of storage (*n* = 3 ± S.D.)

Sample name	t = 0		t = 24 h	
	Z-Ave [nm]	PDI	Z-Ave [nm]	PDI
NE-OC-2.5-US	50 ± 1	0.140 ± 0.009	60 ± 1	0.093 ± 0.009
NE-OC-1.5-US	41 ± 1	0.295 ± 0.050	38 ± 2	0.497 ± 0.044
NE-OH-2-US	53.5 ± 2	0.433 ± 0.019	46.7 ± 2.5	0.491 ± 0.012
NE-OH-1.5-US	55.2 ± 0.5	0.530 ± 0.012	44 ± 2	0.486 ± 0.013
NE-OM-1.5-US	98 ± 3	0.427 ± 0.008	146 ± 5	0.450 ± 0.018
NE-OT-1.5-US	95 ± 2	0.195 ± 0.050	111.7 ± 3	0.407 ± 0.030

As can be seen from the data in Table 3, the nanoemulsions obtained by the low energy emulsification method were characterized by a very small internal phase droplets size, not exceeding 30 nm, and by a polydispersity index ranging from 0.3 to 0.53. On the basis of the results of the measurements carried out after the 24 h storage of the nanoemulsion, the most stable systems were NE-OC-2.5-PIC, NE-OH-2-PIC, NE-OT-1.5-PIC. In these cases, the particle size and PDI did not change significantly over time. The nanoemulsion systems were obtained for each essential oil when the oil:surfactant ratio was 1.5:18.5. Only in the case of the cinnamon oil and tea tree oil, the formulations with higher essential oils concentration were prepared: 2.5% and 2.0% respectively.

The nanoemulsions obtained by the high energy emulsification method (Table 4) were characterized by a small internal phase droplets size within 38–111.7 nm and by polydispersity index in the range of 0.093–0.53. The nanoemulsion containing the cinnamon oil (NE-OC-1.5-US) had the smallest particle size (d = 41 nm) which did not change significantly within 24 h. In this case the oil:surfactant ratio was 1:1 due to the satisfactory results obtained by [34] for the nanoemulsion based on basil oil stabilized with Tween 80 and prepared by the ultrasonification method (Z-Ave = 41, 15, PDI = 0,092).

Regardless of which of the nanoemulsions preparation methods was applied, the smallest droplet size (r = 14 nm and r = 41 nm) was obtained for the cinnamon oil containing the nanoformulations (NE-OC-1.5-PIC and NE-OC-1.5-US, respectively). It may be due to the differences in the oil lipophilicity and in the chemical structure of main oils components (the presence of functional polar or aromatic groups). Pauli [35] tested the lipophilicity (logP) of 25 essential oils. It was determined through the calculation of the lipophilicity (logP) of individual essential constituents and the percentage composition of the essential oil. The cinnamon oil and cassia oil were the essential oils with the lowest lipophilicity. It is believed that lipophilicity of the oil phase significantly influences the size distribution and stability of nanoemulsions. As our previous studies reported, the more hydrophobic oil, the smaller droplet sizes of the nanoemulsions are obtained [36].

The prepared macroemulsions were characterized by a particle size of 2.1–2.5 μm (Table 5). The data in Table 5 indicate the stability of the obtained formulations.

**Table 5:** The mean droplet size of the macroemulsion on the day of preparation and after 24 h and 7 days of storage

Sample name	Mean droplet size [μm] (*n* = 200 ± S.D.)		
	t = 0	t = 24 h	t = 7 days
ME-OC-2.5	2.5 ± 0.1	2.48 ± 0.12	2.53 ± 0.15
ME-OT-1.5	2.42 ± 0.18	2.45 ± 0.23	2.48 ± 0.12
ME-OM-1.5	2.36 ± 0.10	2.33 ± 0.10	2.35 ± 0.10
ME-OH-2.0	2.13 ± 0.10	2.17 ± 0.10	2.26 ± 0.12

The nanoemulsions with unchanged droplet size over 24 h were chosen for the microbiological studies.

### In vitro evaluation of antifungal activity

The results of the antifungal activity of the obtained formulations and four oils against the various fungi strains of pathogenic fungi of plants (*F. culmorum, Ph. cactorum*), dermatophytes (*T. mentagrophytes M. gypseum*) and molds (*S. brevicaulis, A. niger*) are shown in Table 6. The studied concentration for the pure oils ranged from 0.5 to 2.5%. In the case of the nanoemulsions and macroemulsions it was 0.5%.

**Table 6:** Fungicidal activity of the obtained systems

Sample name	Concentration [%]	Inhibition rate of fungi growth (S_g_), % (*n* = 3 ± S.D.)					
		*S.b.*	*A.n.*	*F. c.*	*Ph. c.*	*T.m.*	*M. g.*
OM	2,5	100 ± 0	30 ± 2.65	100 ± 0	100 ± 0	100 ± 0	100 ± 0
OM	1	100 ± 0	0 ± 0	85 ± 4.36	100 ± 0	100 ± 0	100 ± 0
OM	0,5	100 ± 0	0 ± 0	65 ± 5.57	100 ± 0	30 ± 4.59	60 ± 2.0
NE-OM-1,5-US		100 ± 0	40 ± 2.65	95 ± 3.0	100 ± 0	30 ± 1.15	70 ± 1.5
NE-OM-1,5-PIC		69 ± 2.65	0 ± 0	78 ± 2.0	73 ± 4.0	7 ± 0.87	44 ± 6.2
ME-OM-1,5		20 ± 2.65	0 ± 0	10 ± 3.61	13 ± 4.0	0 ± 0	0 ± 0
OC	2,5	60 ± 4.95	15 ± 4.36	58 ± 2.65	100 ± 0	100 ± 0	100 ± 0
OC	1	36±	0 ± 0	38 ± 3.0	100 ± 0	100 ± 0	100 ± 0
OC	0,5	25 ± 3.46	0 ± 0	20 ± 4.16	27 ± 3.6	100 ± 0	100 ± 0
NE-OC-2,5-US	0,5	48 ± 2.0	20 ± 4.36	40 ± 1.53	60 ± 5.5	100 ± 0	100 ± 0
NE-OC-2,5-PIC		8 ± 2.52	0 ± 0	0 ± 0	0 ± 0	53 ± 4.93	100 ± 0
ME-OC-2,5		10 ± 4.36	0 ± 0	0 ± 0	0 ± 0	0 ± 0	0 ± 0
OT	2,5	100 ± 0	25 ± 5.0	100 ± 0	100 ± 0	100 ± 0	100 ± 0
OT	1	53 ± 4.58	0 ± 0	55 ± 6.24	100 ± 0	100 ± 0	100 ± 0
OT	0,5	25 ± 5.0	0 ± 0	25 ± 6.24	30 ± 4.9	55 ± 6.81	100 ± 0
NE-OT-1,5-US	0,5	50 ± 3	0 ± 0	50 ± 5.69	64 ± 1.0	100 ± 0	100 ± 0
NE-OT-1,5-PIC		13 ± 4.58	0 ± 0	20 ± 6.81	8 ± 2.0	18 ± 3.79	35 ± 5.0
ME-OT-1,5		0 ± 0	0 ± 0	15 ± 2.0	0 ± 0	0 ± 0	0 ± 0
OH	2,5	22 ± 3.0	0 ± 0	20 ± 4.93	12 ± 1.0	64 ± 5.57	70 ± 7.0
OH	1	0 ± 0	0 ± 0	15 ± 5.29	8 ± 2.65	20 ± 4.36	25 ± 3.0
OH	0,5	0 ± 0	0 ± 0	0 ± 0	0 ± 0	0 ± 0	0 ± 0
NE-OH-2-US	0,5	0 ± 0	0 ± 0	0 ± 0	0 ± 0	53 ± 5.13	53 ± 7.0
NE-OH-2-PIC		0 ± 0	0 ± 0	0 ± 0	0 ± 0	15 ± 3.46	26 ± 3.6
ME-OH-2		0 ± 0	0 ± 0	0 ± 0	0 ± 0	0 ± 0	0 ± 0

*S. b. Scopulariopsis brevicaulis; A.n. Aspergillus niger; F.c. Fusarium culmorum*; *Ph.c. Phytophthora cactorum*; *T.m. Trichophyton mentagrophytes*; *M.g. Microsporum gypseum*

Significant difference between each treatment and the positive control are shown as *p* < 0.05

## Discussion

**Fig. 3:**
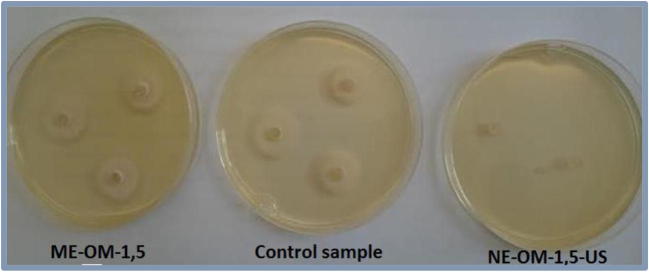
Result of the fungicidal activity of manuka oil-based macroemulsion (ME-OM-1.5), nanoemulsion (NE-OM-1.5-US) and control sample (without the manuka oil) on *F. culmorum* strain

**Fig. 4:**
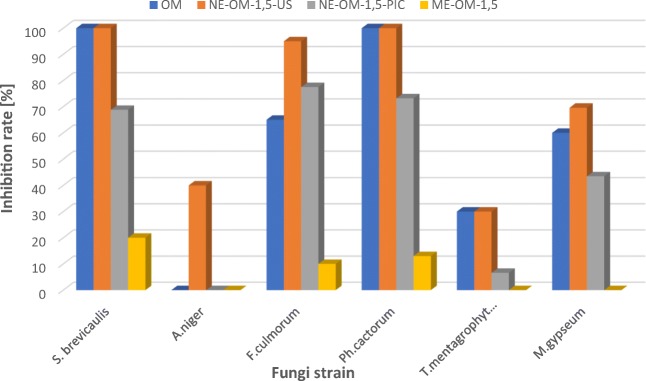
Effect of the pure manuka oil and formulations containing manuka oil on the inhibition rate of fungi growth

Among the formulations containing manuka oil (OM), the best results were obtained for the nanoemulsion prepared with ultrasonification (NE-OM-1.5-US) (Tab.6, Figs. 3 and 4). The nanoemulsions showed excellent fungicidal activity against all types of the tested fungi: one of mold (*S.brevacilus),* both of plant fungi *(F. culmorum, Ph. cactorum*) and one of dermatophytes (*M. gypseum)*. The effect of NE-OM-1.5-US used in the concentration of 0.5% is much better than the pure manuka oil in the same acting dose. It is worth noting that the impact of the nanoemulsion NE-OM-1.5-US on the inhibition rate of fungi growth is similar to the pure manuka oil acting at 5 times higher concentration (2.5%) in relation to all fungi except for *T. mentagrophytes, M. gypseum*.

The advantage of the nanoemulsion lies in the fact that it allows the reduction of the concentration of the active substance (oil) while maintaining biological activity. Moreover, it can improve the essential oil solubility and protect against external factors (e.g. oxidation).

It can also be noticed that the effect of the nanoemulsion based on the essential oils obtained by the Phase Inversion Composition method is less effective than for the samples obtained by the high energy emulsification method. The macroemulsion has low fungicidal action on the *S. brevicaulis* (20% inhibition), *F. culmorum* (10% inhibition), *Ph. cactorum* (13% inhibition) strain. In the case of other fungi, the inhibition rate was equal to 0%.

Similar results were observed for the formulations containing other essential oils (cinnamon, thyme, and tea tree oil). The nanoemulsions obtained by the ultrasonification method based on the cinnamon oil (NE-OC-2.5-US) were characterized by higher antifungal activity against *S. brevicaulis, A. niger, F. culmorum* and *Ph.cactorum* strains in comparison to the pure oil nanoemulsion obtained by the PIC method and the macroemulsion. Also in this case, the nanoformulation NE-OC-2.5-US at the concentration of 0.5% is characterized by a similar fungicidal activity in comparison with the cinnamon oil at the concentration of 2.5% to all tested fungi except for *Ph. cactorum*.

The antimicrobial efficacy of the cinnamon oil nanoemulsions obtained by the low energy and high energy methods was also investigated by Yildirim et al. [29]. However, they did not observe significant differences between spontaneous emulsification and ultrasonification. It may be due to the fact that 10% of surfactant was used to stabilize the nanoemulsions for both methods and similar particle sizes were obtained.

The nanoformulations obtained by the ultrasonification method and based on thyme (NE-OT-1,5-US) oil showed better inhibition of the fungal growth of *S. brevicaulis, F. culmorum, Ph. cactorum, T. mentagrophytes* than the essential oil and other samples in the concentration of 0.5%. Moreover, the nanoemulsion NE-OC-2.5-US at the concentration of 0.5% is characterized by a similar fungistatic action in comparison with the thyme oil at the concentration of 1.0% to all tested fungi except for *Ph. cactorum*.

Both the tea tree oil and the emulsion forms made with it had negligible fungicidal activity in the tested concentration (0.5%). The nanoemulsions acted as a fungicide only against the strains of *T. mentagrophytes* (NE-OH-2-US -53.3%; NE-OH-2-PIC – 15.2%) and *M. gypseum* (NE-OH-2-US *–* 53.2%; NE-OH-2-PIC- 26.1%). The nanoformulation NE-OH-2-US had also comparable fungicidal activity to the pure tea tree oil at the 5-times higher concentration (2.5%) against *T. mentagrophytes* and *M. gypseum* strains.

When comparing the effect of the macroemulsion with nanoemulsions, it can be noticed that the traditional emulsions had very negligible fungicidal activity (10–20% inhibition rate of fungi) or did not show such activity (0% inhibition) against the tested strains.

The *Aspergillus niger* was the most resistant fungi to the essential oil acting at the tested concentration (0.5% *v*/v). Only the nanoemulsions based on the manuka (NE-OM-1.5-US) and cinnamon oil (NE-OC-2.5-US) inhibited the growth of this strain with the rate of 40% and 20%, respectively. The nanoemulsion system based on the manuka essential oil showed the highest activity against the majority of the tested fungal colonies. The effect of these formulation at the concentration of 0.5% *v*/v was similar to the manuka oil which acted at 5-times higher concentration (2.5% v/v).

**Fig. 5:**
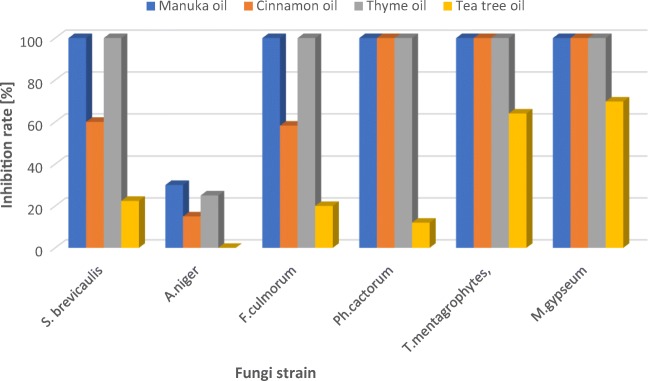
Effect of the pure essential oil on the inhibition rate of fungi growth

Our study showed that the strongest fungostatic activity was found for the manuka and thyme oils, which at 2.5% concentration completely inhibited the growth of the fungi of the species *S. brevicaulis*, *Ph. cactorum*, *F. culmorum*, *M. gypseum* and *T. mentagrophytes* (Fig. 5). Those data are in accordance with the literature reports concerning the activity of thyme oil [13, 14, 18].

The antimicrobial activity of the nanoemulsions is higher than for the free essential oils, which is consistent with the studies of Abd-Elsalam and Khokhlov [25] and Mohamed Ali et al. [17]. In most cases, the nanoemulsions obtained by the ultrasonification method increase the antifungal activity of the used essential oils. It is due to their small droplet size and large surface area which influence the transport of the actives (essential oil) to the cell membrane and their interaction with the multiple molecular sites at the microbial cell membrane. However, it is worth noting that the nanoemulsion obtained by high energy emulsification method was characterized by bigger droplet size (38–111,7 nm) than the formulations obtained by the PIC method (< 30 nm). As was reported in the literature, also Terjung *et. al* [37] obtained an unexpected result, namely, that the emulsions with larger droplet sizes were more effective at inhibiting growth and inactivating cells than with smaller ones. They supposed that this was due to an increased sequestering of antimicrobials on the emulsion interfaces and decreased solubilization in the excess of Tween 80® micelles. Smaller emulsion droplets reduced the antimicrobial activity of the essential oils due to their accumulation at droplet interfaces. Shah et al. [38]. found that free eugenol acted better than its nanodispersed form because there was no binding between the emulsifier and the essential oil which reduced the resulting antimicrobial activity. In the case of our studies, the nanoformulations obtained by ultrasonic emulsification contained much less emulsifier (1.5–2.5%) than those obtained by PIC method (17,5 – 18,5%). It can be the reason why the nanoemulsion prepared by high energy emulsification exhibited greater biological activity despite that they had larger droplet size than the systems prepared by low energy emulsification method.

## Conclusion

In conclusion, it was found that the nanoemulsions prepared by ultrasonification showed excellent fungicidal activity in comparison with the pure oils and macroemulsions. Among others, the nanoformulations of the manuka oil showed the highest activity against the tested fungi. The advantage of nanoemulsion lies in the fact that it allows the reduction of the concentration of the bioactive oils while they maintain antifungal activity. The obtained nanosystems can be applied in pharmaceutical, cosmetic, and agrochemical industries because they increase the antifungal activity of the tested oils against various types of fungi: dermatophytes, molds, and pathogenic fungi of plants.
